# Gene Interactions Regulating Sex Determination in Cucurbits

**DOI:** 10.3389/fpls.2019.01231

**Published:** 2019-10-10

**Authors:** Dandan Li, Yunyan Sheng, Huanhuan Niu, Zheng Li

**Affiliations:** ^1^College of Horticulture and Landscape Architecture, Heilongjiang Bayi Agriculture University, Daqing, China; ^2^College of Horticulture, Northwest A&F University, Yangling, China

**Keywords:** Cucurbitaceae, sex determination, ethylene, gene interaction, transcriptional regulation

## Abstract

The family Cucurbitaceae includes many economically important crops, such as cucumber (*Cucumis sativus*), melon (*Cucumis melo*), watermelon (*Citrullus lanatus*), and zucchini (*Cucurbita pepo*), which share homologous gene pathways that control similar phenotypes. Sex determination is a research hotspot associated with yield and quality, and the genes involved are highly orthologous and conserved in cucurbits. In the field, six normal sex types have been categorized according to the distribution of female, male, or bisexual flowers in a given plant. To date, five orthologous genes involved in sex determination have been cloned, and their various combinations and expression patterns can explain all the identified sex types. In addition to genetic mechanisms, ethylene controls sex expression in this family. Two ethylene signaling components have been identified recently, which will help us to explore the ethylene signaling-mediated interactions among sex-related genes. This review discusses recent advances relating to the mechanism of sex determination in cucurbits and the prospects for research in this area.

## Introduction of Sex Types in Cucurbits

Flower development is the basis of fruit and seed production in plants. In angiosperms, ∼90% of species have perfect flowers with separate stamens and carpels simultaneously. Compared with perfect or bisexual flowers, the differential development or selectivity arrest in the carpel or stamen in some species results in unisexual male or female flowers, respectively, leading to flower sex-type diversity (Tanurdzic and Banks, 2004). Various combinations or distributions of the three kinds of flowers produce hermaphroditic, dioecious, and monoecious plants, thus forming the sex-type diversity of plant. As in animals, the regulation in these floral developmental processes are defined as sex determination or sex differentiation. Sex determination in angiosperms has been well studied in recent years (reviewed by Tanurdzic and Banks, 2004; Lai et al., 2017; Pannell, 2017; Pawełkowicz et al., 2019a).

**Figure 1:**
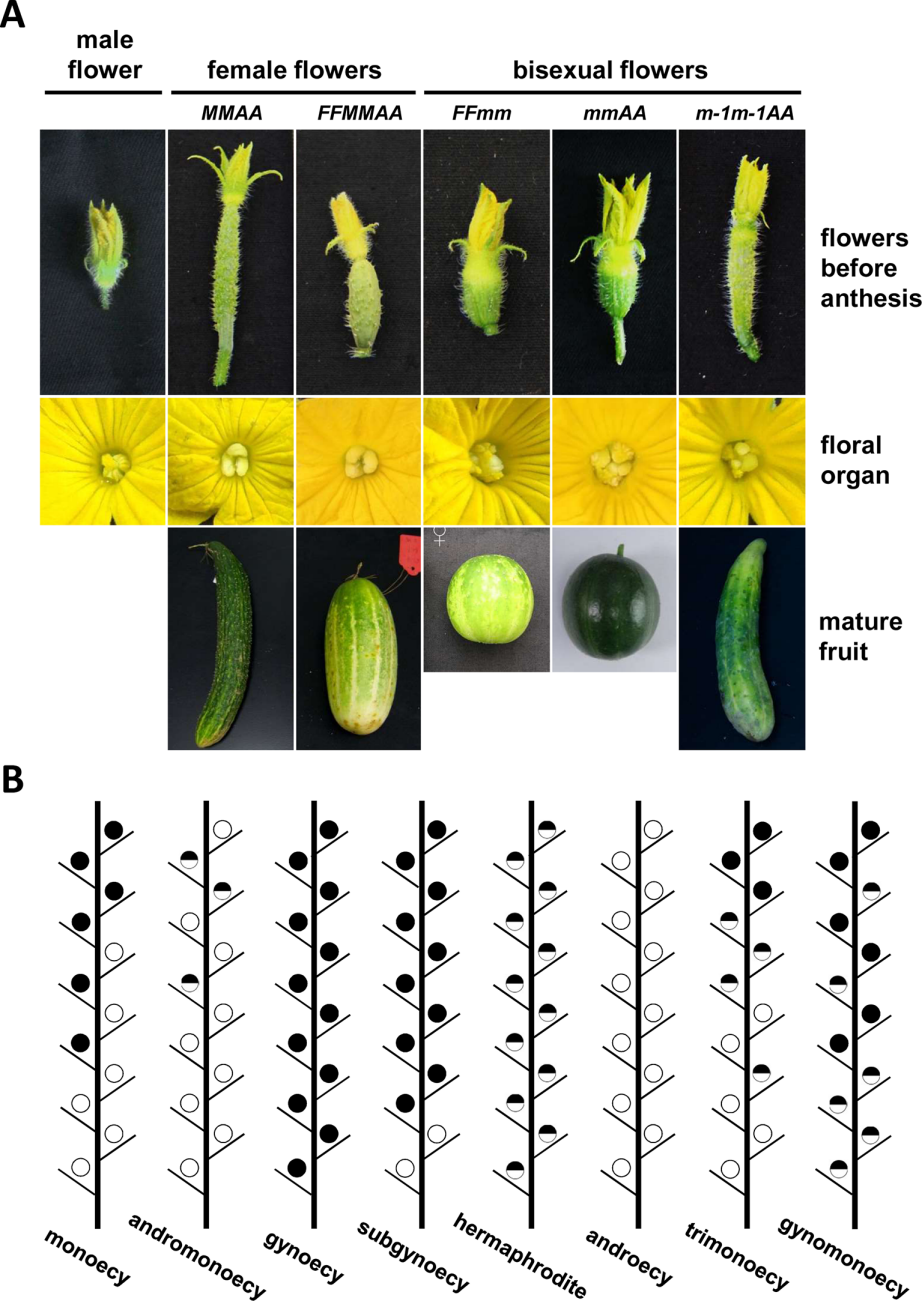
Sex expression in cucurbits. **(A)** Flower sex type and the resulting fruit morphology in five cucumber lines with different genotypes (modified from Tan et al., 2015). **(B)** Schematic diagram of sex expression in the plant main vine in cucurbits. Black, blank, and mix circles represent female, male, and bisexual flowers, respectively. Sex type of the first blooming flower was used to defined the sex type of the node arising the flower.

The family Cucurbitaceae comprises about 120 genera and 960 species, including many economically important crops such as cucumber (*Cucumis sativus*), melon (*Cucumis melo*), watermelon (*Citrullus lanatus*), zucchini (*Cucurbita pepo*), and pumpkins (*Cucurbita moschata*) (Bhowmick and Jha, 2015). The family Cucurbitaceae has abundant flower and plant sex types, and the regulation of sex determination can directly influence their yield and quality. Depending on the distribution or ratio of the three types of flowers produced in a plant (**Figure 1A**), the family Cucurbitaceae is classified into six phenotypes: monoecy, gynoecy, subgynoecy, androecy, andromonoecy, and hermaphrodite (**Figure 1B**). The most common sex type in *Cucumis sativus*, *Cucumis melo*, *Citrullus lanatus*, and *Cucurbita pepo* is monoecy, in which only unisexual flowers bear. However, the distribution of male and female flowers is varied in different species or varieties. Usually, in a monoecious cucumber plant, male flowers arise in early or lower nodes, followed by a mixture of male and female flowers at the middle nodes, and ending with female flowers only in the higher nodes. In cucumber and melon, gynoecious lines produce only female flowers, while androecious plants bear only male flowers. Male and bisexual flowers can be found in andromonoecious lines, which can be regarded as bisexual flowers replacing female flowers found in monoecious lines. Hermaphroditic plants bear only bisexual flowers. Subgynoecious plants, which are found in some watermelon, zucchini, and cucumber lines, produce few male flowers in the beginning nodes and all female flowers in the later nodes. Their most obvious difference from the monoecious lines is the lack of the mixed phase comprising male and female flowers (Galun, 1961; Kubicki, 1969a; Kubicki, 1969b; Kubicki, 1969d). In most cases, bisexual flowers and female flowers are exclusive in a given cucumber and melon plant. However, in a cucumber mutant, certain watermelon plants, and zucchini lines treated with high temperature, hermaphroditic, female, and/or male flowers can arise in the same plant. In these cases, the sex types are named as trimonoecy (or gynomonoecy), trimonoecy, and partial andromonoecy in these three species, respectively (Kubicki, 1969c; Martínez et al., 2014; Ji et al., 2015).

**Figure 2:**
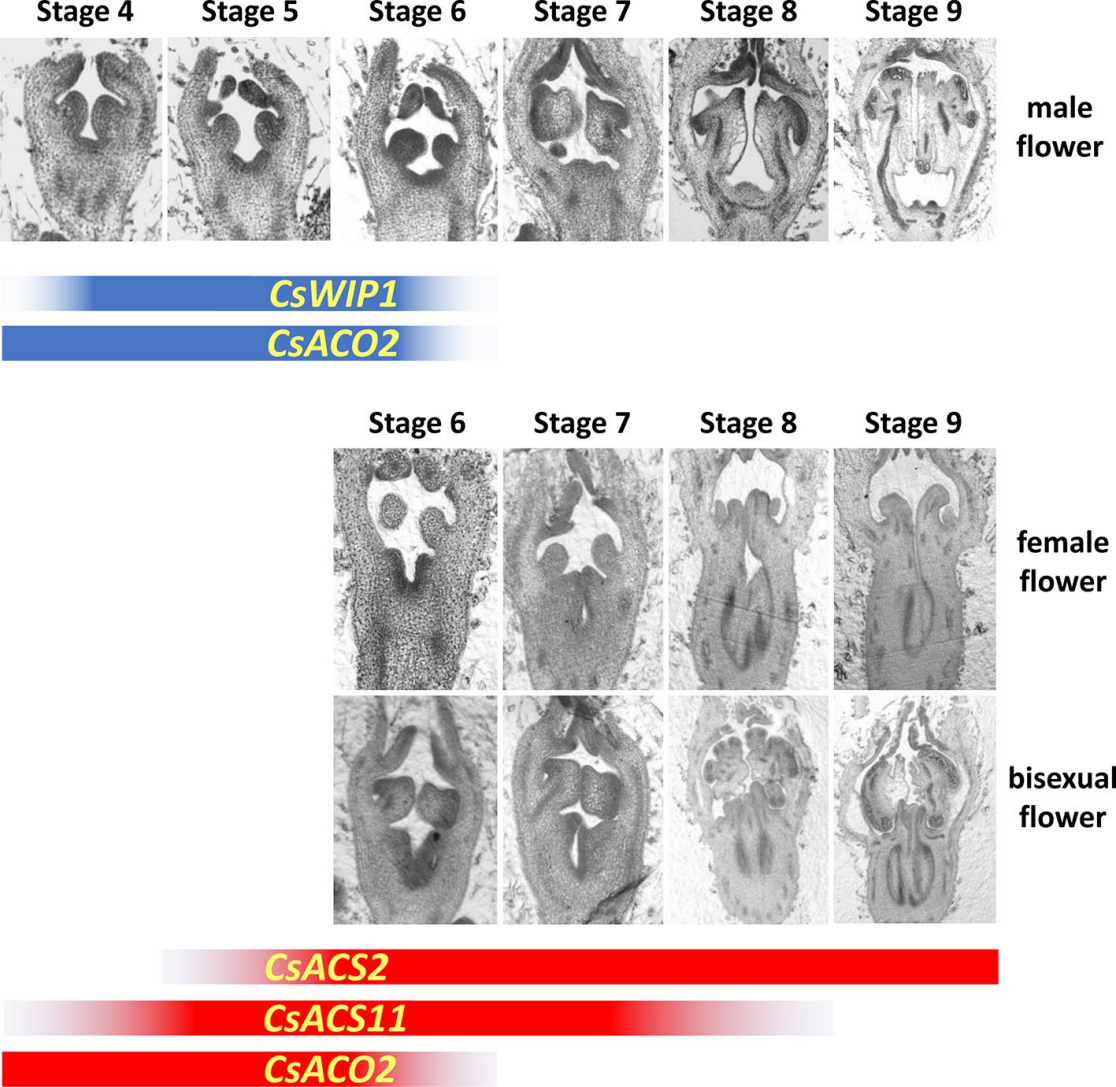
Developmental stages of male, female, and bisexual flowers and the expression pattern of sex-controlling genes in cucumber. Before stage 5, the developmental processes in male, female, and bisexual flowers are similar under visual observation. The detailed developmental program is shown in the text. The expression durations of sex controlling genes are showed in blue (male flower) and red (female flower) bands, and the graduated dark color represents the messenger RNA (mRNA) accumulation. The section assays of male and female flowers are modified from Niu et al., 2018.

In the early stage of flower development, all floral buds are morphologically hermaphrodite, containing staminate and pistillate primordia. Selective arrest of either the staminate or pistillate parts results in female or male flowers, respectively. Furthermore, if the arrest does not happen, bisexual flowers are formed (Atsmon and Galun, 1962). Results of the detailed section assay divided the development of cucumber flowers into 12 stages (Bai et al., 2004). The floral meristem is initiated in stage 1. From stage 2 to 5, sepal, petal, staminate, and pistillate (carpel) primordia are initiated sequentially. Selective arrest then happens after stage 5. In a bud destined to be male, the stamen differentiates anther and filament in stage 6, the anther expands in stage 7, locules differentiate in stage 8, microsporocytes initiate in stage 9, meiosis initiates in stage 10, uninuclear pollen appears in stage 11, and finally, mature pollen is formed in stage 12. In male buds, from stage 6 to 12, the carpel primordia become slightly enlarged. By contrast, in a bud committed to be female, carpel primordia elongate in stage 6, carpel primordia differentiate the stigma and ovary in stage 7, the stigma elongates, and ovule and integument primordia initiate in stage 8, macrosporocytes initiate in stage 9, meiosis initiates in stage 10, embryo sac is formed in stage 11, and finally, all appendant tissues mature in stage 12. The staminate primordia in female flowers can differentiate anthers and filaments in stage 6; however, they are smaller than those in male floral buds. Thereafter, from stage 7, the arrest of stamen development is indicated by their limited size increase. Our data showed that, in bisexual flowers, staminate and pistillate primordia show normal morphological differentiation just as in male and female buds, respectively (**Figure 2**).

Morphologically, after floral meristem initiation, the sex differentiation can be summarized as two options—pistillate initiation, which refers to the induction of pistillate primordia; simultaneous staminate and pistillate primordial growth, which is related to uni/bisexual flower development. Based on these understandings, several gene loci associated with sex expression (sex type) have been identified and cloned in the last two decades. In this review, we discuss recent advances relating to the mechanism of sex determination in the Cucurbitaceae family, beginning with the genes controlling specific processes in sex differentiation.

## Genes Resulting in Pistillate Primordia Initiation

There are two kinds of well-studied gynoecy-controlling gene loci, conferring dominant and recessive gynoecy in cucurbits. The dominant gynoecy is a unique phenotype in cucumber, which is different from other Cucurbitaceous plants. Gynoecy is a particularly important trait in cucumber breeding. Immature cucumber fruit are usually harvested a few days after flowering. Therefore, combined with parthenocarpy, more female flowers mean higher yield in cucumber production. In 1935, Tkachenko described the first gynoecious cucumber in a Japanese (Korean) variety (Kubicki, 1969a). Because there are no male flowers on the gynoecious plants, homozygous gynoecious varieties were nonhereditary before the 1960s, when gibberellic acid (GA) was first used to induce male flowers (Peterson and Anhder, 1960). In 1961, Shifriss explained that a gene (named as *Acr*) could act as an accelerator to impel female flowers to lower nodes (Shifriss, 1961). Later, Galun (1961) and Kubicki (1969a) named the locus as *st* *^F^* and *Acr* *^F^*, respectively. In 1976, the symbol *F* (*Female*) was finally confirmed to represent the dominant gynoecy-controlling locus (Robinson et al., 1976). Later studies indicated that the *F* locus was associated with an additional copy (*CsACS1G*, *G* = gynoecy) of a 1-aminocyclopropane-1-carboxylic acid synthase gene, *CsACS1* (Trebitsh et al., 1997). The open reading frame and proximal promoter (−410 bp upstream sequence) are almost identical between the two genes. The different distal promoter sequence of *CsACS1G* is homologous to that of a putative *branched-chain amino acid transaminase* (*BCAT*) gene (Mibus and Tatlioglu, 2004; Knopf and Trebitsh, 2006). Bioinformatic analysis discovered a copy number variant, which arose from a 30-kb genomic sequence duplication (including *CsACS1* and *BCAT*), involving in the *F* locus. The copy number variant region might present a tandem repeat of the original 30-kb region in gynoecious lines, and the “junction point” of the two repeats is *CsACS1G* (Zhang et al., 2015).

Natural melon and watermelon varieties possess recessive gynoecy loci, which are named as *g* (*g* *ynoecious*) and *gy* (*gy* *noecious*), respectively. Poole and Grimball (1939) reported a recessive *g* locus that controls gynoecy or subgynoecy in melon. Martin et al. (2009) identified the *g* gene as *CmWIP1*, encoding a C2H2 zinc-finger-type transcription factor. Expression of *CmWIP1* leads to carpel abortion, resulting in male flowers. CmWIP1 indirectly represses the expression of *CmACS7*, which is the andromonoecious gene introduced later. In gynoecious lines, an insertion of a transposon (1.3 kb downstream of the gene) represses the expression of *CmWIP1 via* epigenetic changes in its gene promoter. In watermelon, a chromosome translocation produced an insertion mutation in the *ClWIP1* gene (the *CmWIP1* ortholog), leading to a gynoecious line (Zhang et al., 2019). Natural mutants of *CsWIP1* in cucumber varieties are unavailable. Using the clustered regularly interspaced short palindromic repeats/CRISPR-associated protein 9 technology, the created *CsWIP1*-editing mutant lines also showed gynoecy (Hu et al., 2017). All these studies confirmed that WIP1 is a conserved regulator of sex determination in cucurbits.

It should be noted that all the genes controlling gynoecy described above are associated with carpel-bearing flowers. Therefore, the genes function not only in female flowers but also in bisexual flowers. The gynoecy-controlling genes induce (or release) pistillate initiation in female and hermaphroditic flowers.

## Mutant Genes Resulting in the Hermaphroditic Phenotype

Although monoecy is the most common sex type in cucurbits, melon breeders prefer bisexual flowers, making andromonoecy predominant in melon compared with that in other cucurbits (Boualem et al., 2008). Bisexual flowers make natural pollination easier, and the resulting fruits are usually rounder than the products of female flowers (Li et al., 2009; Aguado et al., 2018), both of which are desired phenotypes for melon production. An interesting phenomenon in bisexual flowers is that the fertilization and seed-setting ability in melon is much higher than that in cucumber, which might be the result of long-term domestication in melon breeding.

In cucurbits, the genes controlling the hermaphroditic phenotype are highly conserved. Rosa (1928) stated that andromonoecy is a recessive character in *Cucumis* and *Citrullus*. In melon, the gene locus was named as *a* (*a* *ndromonoecious*) before 2015, then changed to *m* (*m* *onoecious*) to avoid confusion with the androecy controlling gene (*a*). A single-nucleotide mutation in *CmACS7* was identified as associated with andromonoecy in melon. *CmACS7* also encodes a 1-aminocyclopropane-1-carboxylic acid synthase like *CsACS1G*, and the mutation severely loses the enzymatic activity. Expression of *CmACS7* inhibits staminate development in female flowers but is not required for carpel development (Boualem et al., 2008).

Two kinds of natural *monoecious* mutations (named *m* and *m-1*) in cucumber have been identified, and both of which are associated with *CsACS2*, which is an ortholog of *CmACS7* (Boualem et al., 2009; Li et al., 2009; Tan et al., 2015). The mutation in the *m* allele is also a single-nucleotide change; however, it mutates another conserved active site residue, different from the mutation in melon. In addition, a 14-bp deletion is found in the third exon of *CsACS2* in the *m-1* allele, which deduces a truncated protein. The mutations result in severe loss of enzyme activity in plants with the *m* allele and total loss in those with the *m-1* allele.

The *CmACS7* orthologs, *CitACS4*/*ClACS7* and *CpACS27A*, are associated with andromonoecy in watermelon and zucchini, respectively (Martínez et al., 2014; Boualem et al., 2016; Ji et al., 2016; Manzano et al., 2016). In watermelon, the isoforms encoded by *ClACS7* in andromonoecious lines showed no enzymatic activity, and the isoform in the monoecious line was active. In zucchini, even though neither of the parental lines showed a standard andromonoecious phenotype, a mutant nucleotide in *CpACS27A* was considered to be necessary, but not sufficient, to confer partial andromonoecy.

Pleiotropy is another characteristic of the genes controlling the hermaphroditic phenotype. Usually, bisexual flowers produce rounder fruits than female flowers (**Figure 1A**). An interesting finding in cucumber was that the trait of spherical fruit cosegregated with the *m* allele in two large F_2_ populations comprising 5,500 individuals in total (Li et al., 2009). Recently, a study of cucumber fruit growth confirmed that *CsACS2* participates in fruit elongation *via* regulation of ubiquitination (Xin et al., 2019), which supplies a link between sex type and fruit development. In addition, hermaphroditic mutations affect floral organ development. In watermelon, the mutant allele cosegregated with slower growth and maturation of petals and carpels, which resulted in delayed anthesis time in hermaphrodite flowers. Moreover, the number of fruit and seed set were lower in the mutant lines, representing reduced fertilization activity of bisexual flowers in watermelon like in cucumber (Aguado et al., 2018). In zucchini, the bisexual flowers also showed delayed development and maturation of petals and a higher ovarian growth rate (Martínez et al., 2014).

Summarizing the current findings, in cucurbits, the *ACS7* orthologs are expressed in pistil-bearing flowers but not in male flowers. The expression of functional isoforms arrests staminate primordia, producing female flowers, while the nonfunctional or mutant isoforms lose this function and allow staminate and pistillate primordia to grow simultaneously, resulting in bisexual flowers. Therefore, in addition to mutation research, analyzing the regulation of gene expression is also important for the *ACS7* orthologs.

## Mutant Genes Leading to Androecy

In cucumber and melon, sex expression in the main vine and lateral branches are usually different. Usually, the first several nodes in the lateral branch have high feminization potential, producing female flowers in monoecious lines and bisexual flowers in andromonoecious lines. Strictly, a variety without any pistil-bearing flowers in the main vine and lateral branches is defined as androecy; otherwise, it is identified as monoecy (with female flowers) or andromonoecy (with bisexual flowers). Obviously, an androecious line has little economic value in production, and the existing varieties are all mutants. In cucumber, a recessive *a* locus was identified to intensify the androecious nature (Kubicki, 1969b). The gene is hypostatic to the *F* gene, and a plant with a genotype of *ffaa* is completely male. A rare androecious cucumber variety “EREZ” helped to clone the *a* gene, for which the wild-type allele is *CsACS11*, encoding the third 1-aminocyclopropane-1-carboxylic acid synthase involved in sex determination. Similar to the *F* and *M* genes, the mutant isoform isolated from “EREZ” had no enzymatic activity (Boualem et al., 2015). Using a targeting-induced local lesions in genomes strategy, 10 mutations in the melon ortholog of *CmACS11* were created, and two lines containing changes in highly conserved amino acids were observed as androecious.

Besides the traditional *a* locus, an ethyl methanesulfonate-induced mutation helped to discover the second androecious cucumber variety, and the mutation was identified in *CsACO2*, encoding 1-aminocyclopropane-1-carboxylic acid oxidase. The single-nucleotide change in the mutant gene resulted in an inactivated enzyme (Chen et al., 2016). The melon orthologous gene of *CsACO2* is *CmACO3*. Both of these genes showed similar expression patterns (see below).

## Other Sex-Type Related Loci Identified *via* Genetic Analysis

Previous genetic studies have identified many loci that control conventional and accidental sex mutations. In cucumber, besides *F*, *m*, and *a*, the *In* *tensive* *F* *emale* (*In-F*) gene was identified as increasing the female flower ratio in monoecious plants (without the *F* gene). In addition, a plant with both *F* and *In-F* genes could not produce male flowers when treated with GA (Kubicki, 1969b). Kubicki (1969b) also described an *ac* *celerato* *r* gene (*acr* *^1^*), conferring continuous nodes with female flowers in monoecious lines. In subgynoecious cucumber lines, a consistent major quantitative trait locus, which mainly increased the degree of femaleness, was identified on chromosome 3 (*sg3.1*) from two independent studies (Ji et al., 2016; Win et al., 2019). The relationship between *acr* *^1^* or *In-F* and the genes should be clarified in future studies. Using artificial mutagenesis, Kubicki (1974, 1980) identified the *h* *ermaphrodite* (*h*) and *gy* *noecious* (*gy*) loci. Unlike the *m* gene, the *h* gene governs bisexual flowers with normal ovaries as in female flowers, including their shape and pollination ability. However, analysis of the data did not allow us to determine the relationship or difference between the *h* gene and the *m-1* mutation. The recessive *gy* gene was described as intensifying femaleness in cucumber and is linked with the *F* gene. The function of *gy* gene was similar to the *g* gene in melon. However, *CsWIP1* (the melon *g* ortholog in cucumber) resides on chromosome 4, and the *F* gene is on chromosome 6, which means that *gy* and *g* might be two different genes. Trimonoecious plants have been reported in cucumber, watermelon, and zucchini (Kubicki, 1969c; Martínez et al., 2014; Ji et al., 2015). In cucumber, the gene responsible was named as *tr* (*tr* *imonoecious*), while the phenomenon is controlled by the *tm* gene in watermelon (to date, no name has been given in zucchini). However, the detailed structures of bisexual flowers in the three species are different. In cucumber, the bisexual flowers occurring in trimonoecious plants have superior ovaries (hypogynous, the normal bisexual and female flowers are epigynous), derived as a modification of staminate flowers, while the bisexual flowers in trimonoecious watermelon and zucchini seem to be same as those in andromonoecious plants. Unfortunately, standard plant materials possessing the above loci (*In-F*, *acr* *^1^*, *h*, *gy*, *tr*) are not widespread. We look forward to seeing in-depth studies and cloning of these genes in the future.

## Ethylene and Sex Determination in Cucurbits

The most important factor regulating sex expression in cucurbits is the phytohormone ethylene, which controls the transition of female flowering and the ratio of female flowers (Byers et al., 1972; Atsmon and Tabbak, 1979; Owens et al., 1980; Takahashi et al., 1982; Takahashi and Jaffe, 1984; Kamachi et al., 1997; Trebitsh et al., 1997). In cucumber and melon, ethylene (or its releasing agent) has been used to induce female flowers for decades (Rudich et al., 1969; Tsao, 1988; Yin and Quinn, 1995). In zucchini, sex determination in individual floral bud appears to be regulated by ethylene in a similar way (Manzano et al., 2010a; Manzano et al., 2010b; Manzano et al., 2011; Manzano et al., 2013). By contrast, inhibition of ethylene biosynthesis or perception leads to increased maleness in cucumber, melon, and zucchini (Byers et al., 1972; Owens et al., 1980; Manzano et al., 2011). The relationship between ethylene and the sex type in watermelon is complex. In watermelon, female flowers require much more ethylene than male flowers to develop. In addition, bisexual flowers result from a decrease in ethylene production in female floral buds, and ethylene is required to arrest the development of stamens in female flowering, similar to the process in cucumber and melon. Nevertheless, ethylene inhibits the transition from male to female flowering and reduces the number of pistillate flowers, which contrasts with the findings in other cucurbits (Manzano et al., 2014; Zhang et al., 2017a). An interesting phenomenon was observed in watermelon, in which ethephon (an ethylene-releasing reagent) treatment induced numerous abnormal flowers in gynoecious and hermaphroditic plants (Zhang et al., 2017a).

In cucumber and melon, different ethylene responses in staminate and pistillate primordia are used to explain the selective arrest occurring during sex determination. It has been proposed that differing levels of sensitivity in the stamen or carpel primordia could allow each type of primordium to react independently to different ranges of ethylene concentrations (Yin and Quinn, 1995). A higher ethylene threshold for stamen suppression than carpel promotion, coupled with the timing of the increase in ethylene production occurring after the carpels are established, would prevent stamen inhibition before carpel establishment, thereby ensuring the development of flowers (Switzenberg et al., 2014). Ectopic expression of ethylene-related genes suggested that ethylene perception by stamen primordia, but not carpel primordia, is essential for the production of carpel-bearing buds (Little et al., 2007; Switzenberg et al., 2014). Ethylene might promote female flower development *via* an organ-specific induction of DNA damage in primordial anthers. The organ-specific ethylene perception might require downregulation of *CsETR1* (encoding an ethylene receptor protein, see below) expression and increased expression of *CsCaN* (encoding a calcium-dependent nuclease) (Wang et al., 2010; Gu et al., 2011).

Ethylene synthesis results from the activity of 1-aminocyclopropane-1-carboxylic acid (ACC) synthase (ACS) and 1-aminocyclopropane-1-carboxylic acid oxidase (ACO), which transform S-adenosyl-L-Met (SAM) into ACC and convert ACC into ethylene, respectively (Adams and Yang, 1979; Yang and Hoffman, 1984). After biosynthesis, ethylene signaling is perceived by the receptor proteins, which are located in the endoplasmic reticulum. The receptors are negative regulators of ethylene signaling, and in the absence of ethylene, the receptors activate constitutive triple-response 1 (CTR1), which suppresses the ethylene response *via* inactivation of ethylene insensitive 2 (EIN2). Ethylene binding to the receptors switches off the CTR1 phosphorylation activity and activates EIN2. The C terminus of EIN2 is cut and moves into nucleus, stabilizing ethylene insensitive 3/EIN3-like (EIN3/EIL) transcription factors, which can activate the expression of target genes, including those encoding ethylene response factor (ERF) transcription factors. The ERFs then initiate the expression of downstream ethylene-responsive genes (Zhang et al., 2009; Klee and Giovannoni, 2011; Liu et al., 2015).

To date, except for *WIP1* orthologous genes, other sex-controlling genes, including *CsACS1G*, *CsACS2*, *CsACS11*, and *CsACO2* in cucumber, *CmACS7* and *CmACS11* in melon, *CitACS4*/*ClACS7* in watermelon, and *CpACS27A* in zucchini, have important roles in ethylene biosynthesis. Because ethylene participates in sex determination directly in cucurbits, the identification of many sex-related ethylene synthases is not surprising. However, since nearly all the genes show similar biochemical function (producing ethylene), the regulation of their expression should be important. Moreover, a high concentration of ethylene is harmful to young tissue, which was observed in cucumber protoplasts and watermelon plants (Wang et al., 2010; Zhang et al., 2017a). Therefore, the specific spatiotemporal and coordinate expression of the *ACS* and *ACO* genes, which produce local ethylene accumulation, inducing pistil and arresting stamen development, is critical in sex determination.

## Transcriptional Characteristics of the Sex-Related Genes in Sex Determination

Studies with exogenous ethylene have indicated that the timing and concentration are key factors that determine whether carpel or stamen development is affected (Switzenberg et al., 2014). Therefore, the spatiotemporal expression pattern of the sex-related genes should be studied. The developmental process of flower buds reveals that sex determination happens between stage 5 and 6; therefore, all the regulatory genes should function before, or at least no later than, these two periods. *CsACS1G*, *CsACS11*, *CsACS2*, *CmACS11*, *CmACS7*, and *ClACS7*/*CitACS4* are only expressed in female flowers, while *CsWIP1*, *CmWIP1*, and *ClWIP1* are expressed in male flowers. The transcription of *CsACO2* and *CmACO3* has no sex specificity. *CsACS1G* was considered to be autonomously expressed in the shoot or early flower bud before all the other sex-controlling genes in gynoecious cucumber (Knopf and Trebitsh, 2006; Li et al., 2012). However, its detailed expression pattern is still unknown because its low accumulated messenger RNA level limits the use of *in situ* hybridization assays. Transcripts of all the bisexual flower controlling genes, *CsACS2*, *CmACS7*, and *ClACS7*/*CitACS4*, began to accumulate just beneath the pistil primordia of flower buds from stage 5, and then continued to accumulate in central region of the developing ovary (Saito et al., 2007; Boualem et al., 2008; Boualem et al., 2016). The expression signals of both *CsACS11* and *CmACS11* were first detected below the carpel primordia from stage 4 and continued at least until stage 8 (Boualem et al., 2015). *CsACO2* and *CmACO3* expression was first detected in the center of stage 2 to 4 flower buds, just beneath the location of future carpel primordia, and remained expressing in the carpel and stamen at a relatively low levels after stage 6 (Chen et al., 2016). In male flowers, although *CmWIP1* seems to have an enhanced expression compared with *CsWIP1*, they are both expressed from stage 4 to 6 (Boualem et al., 2015; Chen et al., 2016). The expression pattern of sex-controlling genes is summarized in **Figure 2**. The interacting order of these sex-controlling genes in sex determination can be deduced from the sequence and duration of their gene expression.

Ethylene is also a key regulator of the expression of sex-controlling genes. Treatment with exogenous ethylene at an appropriate concentration increased the transcription of *CsACS1*, *CsACS2*, *CsACS11*, *CmACS11*, and *CmACS7* and downregulated that of *CsWIP1* and *CmWIP1* (Yamasaki et al., 2001; Li et al., 2012; Switzenberg et al., 2014; Tao et al., 2018). Endogenous ethylene produced by a first expressed sex-specific gene might also act with other sex-controlling genes, which is used to explain the interacting phenomenon among them (see below). A hypothesis was proposed that ethylene mediated the interaction among different sex-controlling genes, including (to date, at least) *CsACS2*, *CsACS11*, *CmACS11*, *CmACS7*, *CsWIP1*, and *CmWIP1*. Cloning of ethylene signaling factors, CsERF110/CmERF110 and CsERF31, which directly combine with the promoters of *CsACS11*/*CmACS11* and *CsACS2* to activate their expression, respectively, supplied evidence for this hypothesis (Pan et al., 2018; Tao et al., 2018).

Other hormones, like auxin, brassinosteroids (BRs), and GA, are also involved in floral sexual differentiation, all of which might function *via* influencing ethylene biosynthesis or signaling (Rudich et al., 1972; Trebitsh et al., 1987; Yin and Quinn, 1995; Papadopoulou and Grumet, 2005; Zhang et al., 2014a). Auxin increases the expression of *ACS* genes, inducing female flowers (Trebitsh et al., 1987). BRs also increase ethylene production and indirectly participate in cucumber sex determination, in which CsPSTK1 (a putative serine/threonine kinase) might be involved (Papadopoulou and Grumet, 2005; Pawełkowicz et al., 2012). Exogenous GA inhibits ethylene biosynthesis, in which CsGAMYB1 (a GAMYB homolog, positive regulator of GA signaling pathway) and CsGAIP (a DELLA homolog) might have functions (Zhang et al., 2014a; Zhang et al., 2014b).

## Gene Interaction Conferring Sex Expression

**Figure 3:**
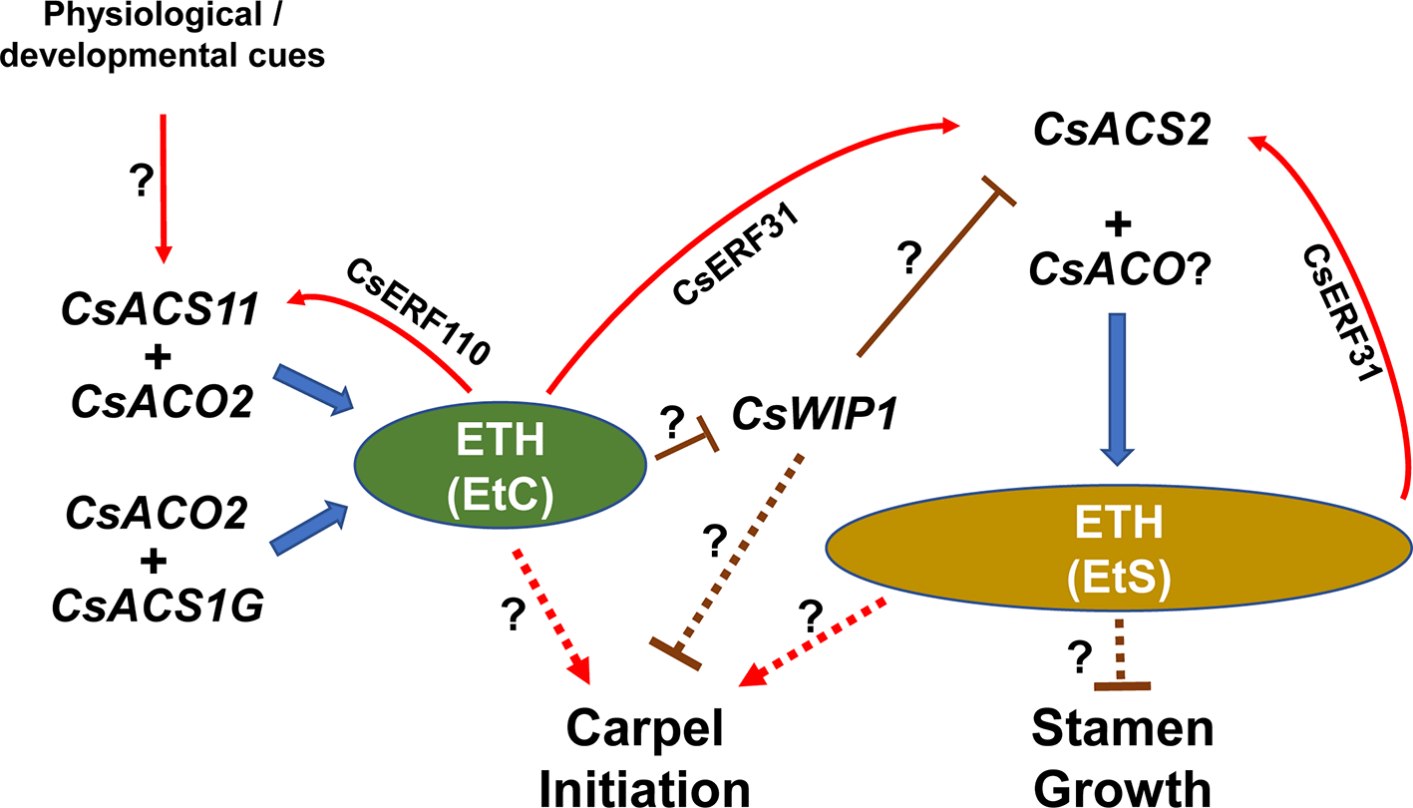
A simplified model outlining major players and interactions in sex determination in cucumber. Transcriptions of *CsACS1G* and *CsACO2* are autonomous, or at least, have no genotype specificity. A red regular arrow indicates directly positive regulation, and “T” represents negative regulation. Dotted lines indicate indirect effects.

Classical genetic analyses helped to propose a systematic phenotype–genotype relationship for each sex type in cucumber, melon, and watermelon (Poole and Grimball, 1939; Galun, 1961; Kubicki, 1969a, Kubicki, 1969b; Kubicki, 1969d; Robinson et al., 1976; Kenigsbuch and Cohen, 1987; Kenigsbuch and Cohen, 1990; Ji et al., 2015). Here, we tried to integrate the results from genetic studies, biochemical assays, and physiological responses (**Figure 3**).

In cucumber, because there are different levels of sensitivity to ethylene in the stamen and carpel, two ethylene thresholds are proposed to define for carpel promotion (EtC) and stamen suppression (EtS), and EtS is believed to be higher than EtC (Switzenberg et al., 2014). Thus, the genotype–phenotype relationship is proposed as:

1. The *F* gene (*CsACS1G*) is autonomously expressed, producing ethylene to reach the EtC (but not higher than the EtS), which initiates pistillate primordia. The ethylene also can induce *M* gene (*CsACS2*) expression, synthesizing higher (and/or long-term) ethylene content reaching the EtS, and arresting staminate primordia. Consequently, a combination of *F* and *M* genes (*FFMM*) produces continuous female flowers and confers the gynoecious phenotype;
2. When the genotype is *FFmm*, EtC can be achieved by *CsACS1G* expression, and the pistil develops normally. However, the mutant *m* gene encodes an inactive ACS, leading to insufficient ethylene to reach the EtS; thus, the stamen develops. Therefore, the *FFmm* genotype results in a plant with all nodes bearing bisexual flowers, producing a hermaphroditic line;
3. When the *F* locus is homozygous recessive (*ff*), the plant sex type is dependent on the expression pattern of the *A* gene (*CsACS11*). If *CsACS11* is expressed, the ethylene becomes higher than the EtC, but lower than the EtS, which can induce pistil initiation and activate *CsACS2* expression, finally producing female flowers. If *CsACS11* is silent, there is insufficient ethylene (< EtC); therefore, the pistillate primordia cannot initiate, while the stamen development is released, resulting in male flowers. Combining these two conditions, the genotype *ffMMAA* results in a monoecious individual;
4. Like the phenomenon in hermaphroditic lines, when the *m* locus is homozygous recessive, stamens in female flowers are not suppressed, and genotype *ffmmAA* results in andromonoecious plants;
5. When both of *F* and *A* loci are mutated (*ffaa*), no genes can produce ethylene up the level of the EtC, in which case *CsACS2* and pistillate primordia cannot be induced. Therefore, only male flowers can develop, and an androecious plant emerges (Li et al., 2012; Tao et al., 2018).

Because no natural *CsWIP1* and *CsACO*2 mutations were used in these previous genetic analyses, all the plants studied were assumed to have wild-type *CsWIP1* and *CsACO2* genes. Analysis in melon showed that *CmWIP1* negatively regulates the expression of *CmACS7* (its ortholog in cucumber is *CsACS2*) (Martin et al., 2009). Ethylene could downregulate the expression of *CsWIP1*, meaning that the EtC may induce *CsACS2 via *suppressing *CsWIP1*. In a mutant *Cswip1* background, the expression of *CsACS2* is released, producing the EtS directly, which is enough to initiate pistillate primordia and arrest staminate primordia, resulting in female flowers and gynoecious plants (Hu et al., 2017). In addition, CsWIP1 might directly suppress pistillate primordia initiation *via* an unknown pathway, which was proposed in melon (Boualem et al., 2015). The enzyme encoded by *CsACO2* is considered to at least combine with *CsACS1G* and *CsACS11* to complete ethylene synthesis. Therefore, *Csaco2* mutants break the formation of EtC, resulting in an androecious phenotype (Chen et al., 2016). In the future, the sex type of the *Cswip1Csaco2* double mutant should be investigated to identify the relationship between *CsACS2* and *CsACO2* in cucumber.

In melon, except for the dominant *F* gene, all the sex-type-related genotype–phenotype relationships are similar to those in cucumber. Therefore, plant femaleness is dependent on mutant *Cmwip1* (the *g* gene in melon) and/or *CmACS11* expressing (the *A* gene in melon). The bisexual flowers in melon are the result of mutations in *CmACS7* (the *m* gene in melon). Classical genetics confirmed that the genotype *MMAAGG* results in a monoecious plant, *mmAAGG* results in andromonoecy, *MMgg* results in gynoecy, *mmgg* results in a hermaphrodite, and *aaGG* results in androecy (Poole and Grimball, 1939; Kenigsbuch and Cohen, 1987; Kenigsbuch and Cohen, 1990). An interestingly different phenomenon was observed between monoecious cucumber and melon plants with the same (*ff*)*MMAA*(*GG*) genotype that femaleness on the main vine of cucumber (although it is often changeable) is higher than that in melon (usually all nodes produce male flowers). This might refer to different expression activity of the *A* genes in these two species. We have identified that ethylene could induce the expression of *CsACS11* (Tao et al., 2018). However, there is no *CsACS1G* in monoecious lines to autonomously produce ethylene. Therefore, other physiological or developmental cues inducing *CsACS11* or *CmACS11* should be identified in future studies, which might help to explain the problem stated by Ma and Pannell (2016) that “what decides whether *ACS11* is on or off in particular flowers.”

In watermelon, three recessive alleles were suggested to control the sex types: *a* *ndromonoecious* (*a*), *gy* *noecious* (*gy*), and *t* *ri* *m* *onoecious* (*tm*) (Ji et al., 2015). Therefore, phenotype–genotype relationships are proposed as: monoecious, *AAGyGyTmTm*; trimonoecious, *AAGyGytmtm*; andromonoecious, *aaGyGy*; gynoecious, *AAgygyTmTm*; gynomonoecious, *AAgygytmtm*; and hermaphroditic, *aagygy*. Recent gene cloning helped to identify that the *a* gene is a mutation in *ClACS7*/*CitACS4*, and the *gy* gene is mutated in *ClWIP1*, which are orthologous genes of *CsACS2*/*CmACS7* and *CsWIP1*/*CmWIP1*, respectively (Boualem et al., 2016; Ji et al., 2016; Manzano et al., 2016;Zhang et al., 2019). Therefore, it seems that the *WIP1*-*CsACS2*/*CmACS7* relationship is conserved in all the studied cucurbits.

There is little information about the phenotype–genotype relationship in zucchini. Sex determination in individual floral buds of zucchini appears to be regulated by ethylene in the same way as that in melon and cucumber, and the *ACS7* ortholog *CpACS27A* is also involved in bisexual flower development (Manzano et al., 2010a; Manzano et al., 2010b; Manzano et al., 2011; Martínez et al., 2014). Therefore, we believe that the conserved *ACS11*-*WIP1*-*CsACS2*/*CmACS7*/*CpACS27A* pathway also exists in zucchini.

## Suggestion for Gene Nomenclature

An imminent work is unifying gene names controlling the similar sex types in cucurbits. For example, the abbreviated names of *andromonoecious* (which is now *m*, but used to be *a* before 2015) and *androecious* (is now *a*) in melon, easily cause confusion. Moreover, the genotype symbols in watermelon are also liable to cause misunderstanding. Because all the genes controlling the appearance of bisexual flowers are *Arabidopsis* *ACS7* orthologs, we suggest that the symbol *m* is used for the andromonoecious phenotype, just as in cucumber and melon. Likely, the symbol *g* is suggested to use for the recessive gynoecy produced by *wip1* ortholog mutation. The structures of bisexual flowers in cucumber trimonoecious mutant (hypogynous) and in trimonoecious watermelon (seemingly normal) are different. Therefore, it is necessary to retain the current gene nomenclature (*tr* and *tm*).

Another gene symbol that needs to be discussed is *f* in cucumber. When we reexamined the *F* gene and its genomic structure, it was clear that the *F* locus is unique in gynoecious cucumber lines with a tandem 30-kb repeat (Zhang et al., 2015). This locus does not exist in lines with only one copy of the 30-kb region, as in monoecious, andromonoecious, and androecious lines. Previously, genotypes of these latter three lines were usually written as homozygous *ff* for this locus, which is used to represent the recessive allele in the *F* locus. However, we now know that there is only one form of gene (*CsACS1G*) in this locus, and no studies have demonstrated a mutant or a nonfunctional allele. In traditional cognition, the dominant *F* gene is *CsACS1G*, and the recessive *f* is considered as *CsACS1*. However, this is not right. All cucumber lines tested have *CsACS1*, and only gynoecious plants possess both *CsACS1* and *CsACS1G*. These findings mean that *CsACS1* and *CsACS1G* are not alleles of the same gene, which are not located in a same locus (30 kb apart approximately). Therefore, at this time, before a *CsACS1G* mutation is discovered, we suggest that the nomenclature of the *f* gene makes no sense and should be omitted in the genotype.

Consequently, we suggest the phenotype–genotype relationships in cucurbits as: monoecious, *MMAAGG*; andromonoecious, *mmAAGG*; androecious, *aaGG*; gynoecious, *FFMM* (in cucumber only) or *MMgg*; hermaphroditic, *FFmm* (in cucumber only) or *mmgg*; trimonoecious, *MMAAGGtrtr* in cucumber and *MMGGtmtm* in watermelon; gynomonoecious, *FFMMtrtr* in cucumber and *MMggtmtm* in watermelon.

## Other Aspects Relating to Sex Type

Detailed descriptions about many of the transcriptomic, epigenomic, and metabolomic research related to sex type are beyond the scope of this manuscript (Miao et al., 2011; Wang et al., 2014; Gao et al., 2015; Zhang et al., 2017b; Lai et al., 2017; Latrasse et al., 2017; Lai et al., 2018a; Lai et al., 2018b; Song et al., 2018; Wang et al., 2018; Zhou et al., 2018; Wang et al., 2019; Pawełkowicz et al., 2019b). The sex-related genes and cues identified in these studies are associated with temperature, photoperiod, blue/red light, hormone synthesis and signaling, lipid and sugar metabolism, the cell cycle, etc. However, we do not know whether the genes are causes or results of the sex-type changes, and we cannot summarize the accurate locations of these genes in the gene pathway of sex determination.

## General Characteristics of Sex Controlling Genes

It is not surprising that near all the sex controlling genes are “ethylene synthases.” Therefore, the expression regulation of each gene should be conducted, and the specific transcription regulators for a given sex-control gene should be identified. In addition, exploring new sex-related mutations has always been a priority in sex determination research. Considering all the known genes that directly control the sex types in cucurbits, we propose that a sex-related gene may have more than one of the following characteristics: (1) the gene product directly participates in ethylene synthesis or signal transduction, (2) the gene or its product directly or indirectly regulates a known sex-control gene, (3) gene expression responds to ethylene or the factors interfering with ethylene synthesis or signaling, and (4) critically, mutation of the gene can change sex type. We believe that these characteristics could help to identify new sex-related genes in the future.

## Future Prospects

The mechanism of sex determination is of great interest to researchers. Meanwhile, the close relationship between sex type and yield in cucurbits has attracted increased attention in plant breeding. At present, a model of the ethylene core has been established in four cucurbit species (cucumber, melon, watermelon, and zucchini). However, the direct regulators and the molecular details remain poorly understood. Exploring more mutations and using reverse genetics are the most effective way to identify a gene-controlling sex differentiation. In addition, since the critical developmental stage in sex determination is clear, more precise approaches, such as laser microdissection and single-cell RNA sequencing have the potential to reveal the detailed gene pathways involved in this process. We hope that the suggestions proposed in this review are conducive to revealing the mechanisms of sex determination in cucurbits.

## Author Contributions

DL and YS collected and organized the references. HN supplied the results of section assay. ZL conducted the figures. DL and ZL wrote the paper.

## Funding

This work was supported by the National Natural Science Foundation of China (31672150 and 31872111 to ZL, and 31772330 to YS), the National Science Foundation of Heilongjiang Province (QC2016035), the Innovative Talen Program of Heilongjiang Bayi Agriculture University (2016-KYYWF-0164) (to DL), the Fundamental Research Fund for the Central Universities (2452016004), the Sci-Tech New Star Program of Shaanxi Province (2017KJXX-57), and Key Research and Development Plan (2018NY-034) of Shaanxi Province (to ZL).

## Conflict of Interest

The authors declare that the research was conducted in the absence of any commercial or financial relationships that could be construed as a potential conflict of interest.
